# Whole-Exome Sequencing Identifies a Novel Germline Variant in *PTK7* Gene in Familial Colorectal Cancer

**DOI:** 10.3390/ijms23031295

**Published:** 2022-01-24

**Authors:** Beiping Miao, Diamanto Skopelitou, Aayushi Srivastava, Sara Giangiobbe, Dagmara Dymerska, Nagarajan Paramasivam, Abhishek Kumar, Magdalena Kuświk, Wojciech Kluźniak, Katarzyna Paszkowska-Szczur, Matthias Schlesner, Jan Lubinski, Kari Hemminki, Asta Försti, Obul Reddy Bandapalli

**Affiliations:** 1 Molecular Genetic Epidemiology, German Cancer Research Center (DKFZ), 69120 Heidelberg, Germany; b.miao@kitz-heidelberg.de (B.M.); mando.skopelitou@yahoo.de (D.S.); srivastava.aayushi97@gmail.com (A.S.); sara.giangiobbe@gmail.com (S.G.); abhishek@ibioinformatics.org (A.K.); a.foersti@kitz-heidelberg.de (A.F.); 2 Hopp Children’s Cancer Center (KiTZ), 69120 Heidelberg, Germany; 3 Division of Pediatric Neurooncology, German Cancer Research Center (DKFZ), German Cancer Consortium (DKTK), 69120 Heidelberg, Germany; 4 Medical Faculty Heidelberg, Heidelberg University, 69120 Heidelberg, Germany; 5 Department of Genetics and Pathology, Pomeranian Medical University, 71252 Szczecin, Poland; dagmara.dymerska@gmail.com (D.D.); magdalenakuswik@gmail.com (M.K.); dante.k@wp.pl (W.K.); katarzynapredygier@yahoo.pl (K.P.-S.); lubinski@pum.edu.pl (J.L.); 6 Computational Oncology, Molecular Diagnostics Program, National Center for Tumor Diseases (NCT), 69120 Heidelberg, Germany; n.paramasivam@dkfz-heidelberg.de; 7 Institute of Bioinformatics, International Technology Park, Bengaluru 560066, India; 8 Manipal Academy of Higher Education (MAHE), Manipal 576104, India; 9 Bioinformatics and Omics Data Analytics, German Cancer Research Center (DKFZ), 69120 Heidelberg, Germany; matthias.schlesner@informatik.uni-augsburg.de; 10 Faculty of Medicine and Biomedical Center in Pilsen, Charles University in Prague, 30605 Pilsen, Czech Republic

**Keywords:** colorectal cancer, *PTK7*, germline variant, AKT signaling pathway, familial cancers, familial cancer variant prioritization pipeline

## Abstract

Colorectal cancer (CRC) is the third most frequently diagnosed malignancy worldwide. Only 5% of all CRC cases are due to germline mutations in known predisposition genes, and the remaining genetic burden still has to be discovered. In this study, we performed whole-exome sequencing on six members of a Polish family diagnosed with CRC and identified a novel germline variant in the protein tyrosine kinase 7 (inactive) gene (*PTK7*, ENST00000230419, V354M). Targeted screening of the variant in 1705 familial CRC cases and 1674 healthy elderly individuals identified the variant in an additional familial CRC case. Introduction of this variant in HT-29 cells resulted in increased cell proliferation, migration, and invasion; it also caused down-regulation of CREB, p21 and p53 mRNA and protein levels, and increased AKT phosphorylation. These changes indicated inhibition of apoptosis pathways and activation of AKT signaling. Our study confirmed the oncogenic function of *PTK7* and supported its role in genetic predisposition of familial CRC.

## 1. Introduction

Colorectal cancer (CRC) is the third most frequently diagnosed malignancy worldwide, accounting for 10.2% of all cancer cases in 2018. Although incidence as well as mortality rates have been reported to stabilize or even decrease in highly developed countries, they still account for a large share of cancer diagnoses and deaths in the world [1,2]. Since an important approach to cancer prevention targets the improvement of screening and early diagnosis methods, understanding the heterogeneous etiological background of CRC, including environmental and inherited genetic factors, is of great relevance. Although up to 35% of CRC susceptibility is considered to be explained by heritability and around 15% of CRC patients have a family history of CRC, less than 5% of all cases are due to highly penetrant germline variants in established predisposition genes, such as *APC,* mismatch repair genes (*MLH1*, *MSH2*, *MSH6*, *PMS2*), *EPCAM*, *SMAD4*/*BMPR1A,* and *MUTYH*, resulting in well-characterized clinical features of known Mendelian CRC syndromes [3,4,5]. Recent studies using next generation sequencing techniques have proposed novel predisposing genes such as *NTHL1*, *RNF43*, *POLE*, *POLD1*, *FAN1*, and *RPS20* contributing to inherited CRC susceptibility [6,7,8,9,10,11,12,13]. Nevertheless, the remaining genetic burden of familial CRC has still not been sufficiently explored and may reveal novel highly penetrant germline variants.

With the aim of identifying such potential CRC predisposing variants, we performed germline whole-exome sequencing (WES) on a family from Poland with multiple individuals affected by CRC. For candidate identification, we applied a pedigree-based study design with integrated bioinformatics and functional analysis strategy developed by us [14,15]. We identified a novel missense variant in the *PTK7* gene (ENST00000230419, V354M) involved in multiple cellular processes such as proliferation, apoptosis, cell adhesion, polarity, and migration. The encoded protein tyrosine kinase 7 further acts as a molecular switch in multiple signaling pathways such as Wnt or VEGF signaling pathways, potentially explaining its frequent deregulation in cancer [16,17,18].

## 2. Results

### 2.1. Prioritization of a Novel PTK7 Missense Variant Using FCVPPv2

**Figure 1:**
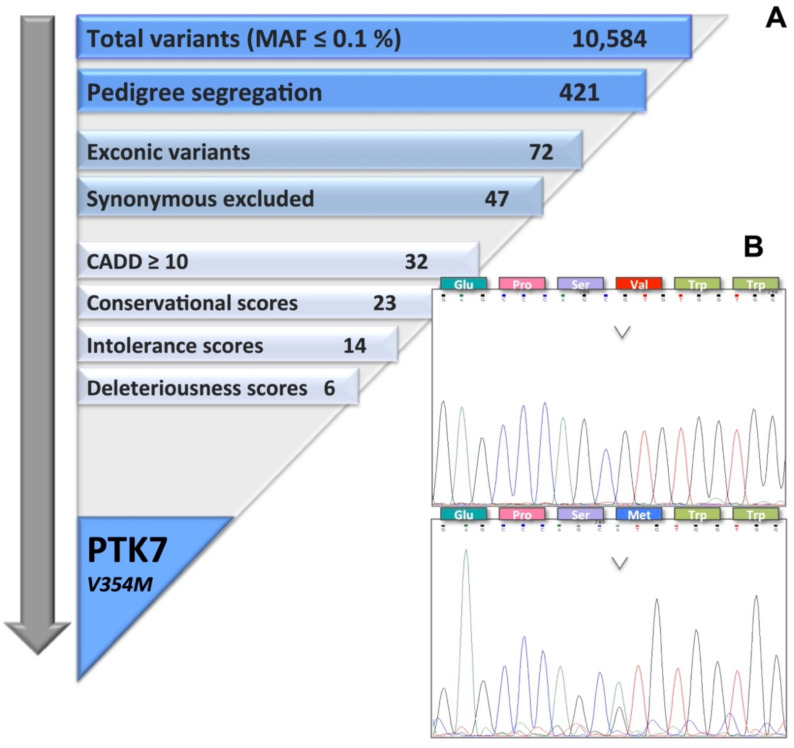
Prioritization of the missense variant V354M in the *PTK7* gene. (**A**) Flow chart displaying the filtering process of exonic variants according to the FCVPPv2. Out of 10,584 identified variants, the missense variant V354M in *PTK7* gene was prioritized. *CADD*–*combined annotation-dependent depletion tool*. (**B**) Electropherograms representing the wild type *PTK7* sequence (upper panel) identified in family members II-6 and III-2 and the heterozygous *PTK7^V354M^* variant (lower panel) identified in family members I-2, II-1, II-2, and II-4. The respective substitution Val → Met is displayed in the amino acid sequences. The color of curves indicates the detected nucleobases: black stands for guanine, green for adenine, blue for cytosine, and red for thymine. *Glu*–*glutamic acid*, *Pro*–*proline*, *Ser*—*serine*, *Val*—*valine*, *Trp*—*tryptophan*, *Met*—*methionine*.

Processing of the WES data through variant calling, annotation, and filtering with a MAF ≤ 0.1% revealed a total number of 10,584 variants (Figure 1A). Pedigree segregation criteria narrowed this number down to 421 variants. Most of the variants were located in the intergenic or intronic regions, leaving 72 coding variants for further evaluation. Exclusion of synonymous variants due to a potentially less deleterious nature resulted in 47 non-synonymous, frameshift deletion, and exonic variants of unknown classification, of which 32 were annotated with a PHRED-like CADD score of ≥10. Conservational screening reduced this number to 23 variants, intolerance screening furthermore to 14, and deleteriousness screening lastly to 6 final exonic variants after passing through all steps of our in-house developed variant prioritization pipeline: *ADAMTS10* (V104M), *C2orf42* (S459R), *GNA13* (I54V), *RSBN1L* (R141H), *TNIP1* (E238K), and *PTK7* (V354M) (Figure 1B, Table 1).

The top-listed variants were further assessed by the latest version of the gnomAD Browser [20], CGI [21], cBioPortal [22], and recent literature. All final exonic variants were predicted as passenger mutations by CGI with gnomAD allele frequencies ≤ 0.1% in the non-Finnish European (NFE) population. Based on TCGA PanCancer Atlas data (NIH, Bethesda, MD, USA) comprising 594 colorectal adenocarcinoma samples, *PTK7* and *ADAMTS10* genes showed the highest overall somatic alteration frequency of around 4%, suggesting these two genes to be the most likely candidates for the development of CRC in the studied family. A recent study identified frequent somatic mutations in the *ADAMTS10* gene, but the gene was an unlikely candidate for germline predisposition, as it lacked any other associations with colorectal carcinogenesis [23]. In contrast, overexpression of the PTK7 protein has been widely reported in several malignancies, particularly in CRC. Based on all this evidence and in silico predictions, we prioritized the identified variant in the *PTK7* gene in our FCVPPv2 analysis.

### 2.2. Confirmation of Familial Segregation and Identification of an Additional Carrier of the PTK7^V354M^ Variant

Targeted Sanger sequencing for exon 7 of the *PTK7* gene showed the heterozygous variant *PTK7*^V354M^ in the affected family members I-2, II-1, and II-4 and in one member with polyps II-2. The other family member with polyps II-6 had the wild-type sequence, as did the healthy individual III-2, confirming the pedigree segregation of the variant (Figure 1B). Targeted genotyping of 1704 Polish unrelated familial CRC cases and 1674 healthy elderly individuals, using custom-made Taqman assay, identified the *PTK7*^V354M^ variant in an index case of a CRC family, who was diagnosed with CRC at the age of 66 years; his father had a CRC diagnosis at the age of 68 years, and his half-sibling from the father’s side had CRC at the age of 30 years. No variants were found in controls.

**Figure 2:**
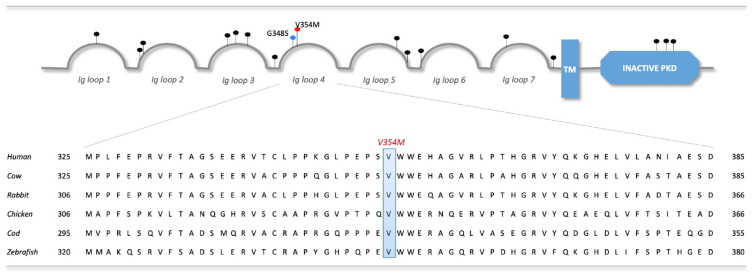
Localization of PTK7 mutations in CRC on protein level and mapping of the missense variant V354M to multiple sequence alignments of PTK7. All PTK7 somatic mutations identified in colorectal adenocarcinoma were extracted from cBioPortal (www.cbioportal.org) on the 15 March 2020 using TCGA PanCancer data and are marked by black pins. Germline variants are represented by colored pins, blue for the G348S variant in neural tube defects and red for the V354M variant identified in this study. The induced amino acid substitution V354M affects the Immunoglobulin (Ig) loop 4 domain (pp. 309–407) and is mapped to multiple protein sequence alignments of PTK7 for selected species. PTK7 sequences were downloaded from Ensembl (GRCh37/hg19) [24] for human (ENSG00000112655), cow (ENSBTAG00000012761), rabbit (ENSOCUG00000002874), chicken (ENSGALG00000008609), cod (ENSGMOG00000011589), and zebrafish (ENSDARG00000011863). As demonstrated, the variant affects an amino acid residue identically in all sequences and thus is highly conserved. *TM*—*transmembrane domain*, *PKD*—*protein kinase domain*.

In order to assess the potentially activating amino acid substitution induced by the variant, multiple protein sequences of PTK7 were extracted from Ensembl (GRCh37/hg19, EMBL-EBI, Hinxton, UK) and multiple sequence alignment was performed (Figure 2) [24]. The induced amino acid substitution (V345M) was predicted to affect the immunoglobulin (Ig)-like C2-type 4 domain (pp. 309–407) encoding for one of the seven extracellular lg loops, according to UniProt data (EMBL-EBI, Hinxton, UK; SIB, Lausanne, Switzerland; PIR, Washington, DC, US, #Q13308) [25]. Due to the high sequence conservation across the species (human, cow, rabbit, chicken, cod and zebrafish), a functional importance is assumed for the affected region. Analysis with cBioPortal further revealed 15 somatic mutations affecting PTK7 protein identified within 594 colorectal adenocarcinoma samples from TCGA PanCancer Atlas data (frequency = 2.36%). The overall somatic alteration frequency of PTK7, including copy number alterations, was calculated to reach about 3.54% for CRC and up to 10% for other cancers (Supplementary Figure S1) [22,26]. Additionally, germline variants affecting the *PTK7* gene have been reported in neural tube defects including the adjacently located missense variant G348S [27].

### 2.3. PTK7^V354M^ Variant Increases PTK7 Protein Levels by Potentially Altering Protein Stability

**Figure 3:**
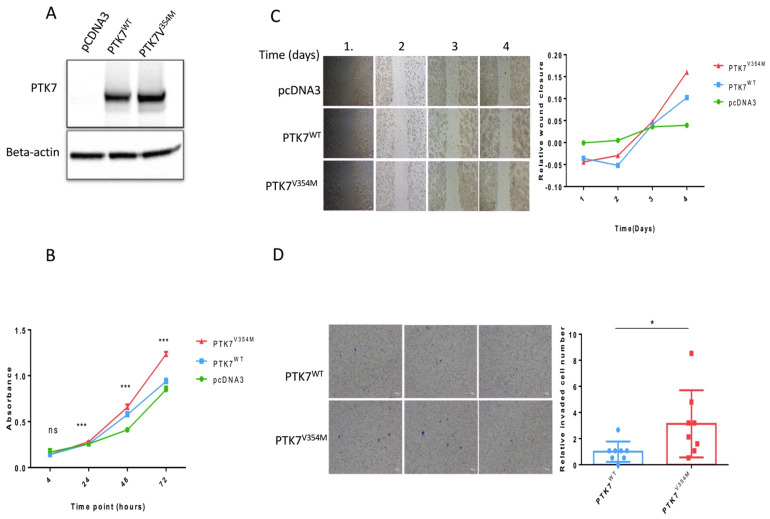
*PTK7*^V354M^ variant increases cell proliferation, migration, and invasion. (**A**) Western blot results represent enhanced protein expression of PTK7 in HT29-*PTK7^V354M^* cells compared to HT29-*PTK7*^WT^ cells. (**B**) Proliferation assays show significantly increased viable cell numbers of HT29-*PTK7*^V354M^ compared to HT29-*PTK7*^WT^ cells. Significance levels are included for each time point. ns—no significance, *** *p* < 0.001. (**C**) Representative image of migrated HT-29 cells after overexpression of *PTK7^WT^ and PTK7^V354M^*. (**D**) Representative image of invaded HT-29 cells after overexpression of *PTK7^WT^* and *PTK7^V354M^*. * *p* < 0.05. *PTK7^V354M^–PTK7 plasmid with the mutation V354M, PTK7^WT^–wild type PTK7 plasmid, pcDNA3–control vector*.

With the aim of investigating the functional impact of the variant, Western blots targeting PTK7 protein were conducted. Results show higher protein expression of PTK7 in HT29-*PTK7*^V354M^ compared to HT29-*PTK7*^WT^ cells, indicating an enhancing impact of the introduced variant on protein expression level, while HT29-pcDNA3 cells did not express PTK7 protein at all, as reported in Harmonizome [28] (Figure 3A).

### 2.4. PTK7^V354M^ Variant Enhances Cell Proliferation in Human Colon Cancer Cells

Cell proliferation assays resulted in significantly increased viable cell numbers of HT29-*PTK7*^V354M^ compared to HT29-*PTK7*^WT^ cells at all measured time points starting from day 1. HT29-pcDNA3 cells showed the lowest proliferation curve, confirming the proliferative impact of PTK7 protein in general and the introduced variant in particular (Figure 3B).

### 2.5. PTK7^V354M^ Variant Increases Invasion and Migratory Properties of Cells

The effect of PTK7^V354M^ variant on cell migration measured using wound stimulus by time-lapse scanning microscopy showed that HT-29 cells transfected with PTK7^V354M^ were able to close the wound faster than cells transfected with PTK7^WT^ (Figure 3C). In the invasion assay, the total number of invasive cells counted using a microscope was significantly higher in PTK7^V354M^ transfected cells compared to PTK7^WT^ transfected cells (Figure 3D).

### 2.6. Suppression of p53, p21 and CREB Gene Transcription Induced by the PTK7^V354M^ Variant

**Figure 4:**
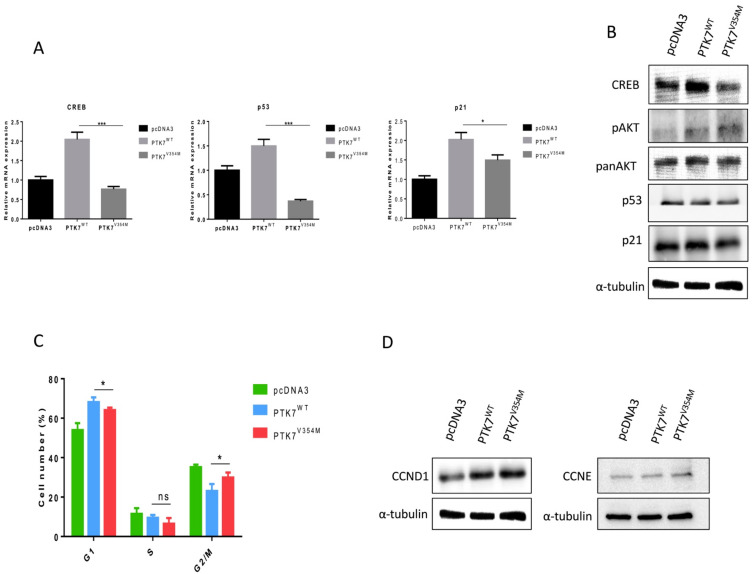
Effect of *PTK7*^V354M^ variant on downstream targets and cell cycle. (**A**) qPCR resulted in significant down-regulation of *p53, p21*, and *CREB* mRNA levels in HT29-*PTK7^V354M^* cells. * *p* < 0.05, *** *p* < 0.01. (**B**) Western blot results representing enhanced protein expression of pAKT as well as decreased CREB, p53 and p21 protein expression in HT29-*PTK7^V354M^* cells compared to HT29-*PTK7*^WT^ cells. (**C**) HT29-PTK7 ^V354M^ transfected cells resulted in significant cell cycle progression with less accumulation of cells in the G0/G1 phase compared to HT29-*PTK7*^WT^ cells and a concomitant increase in G2/M phase, confirming the cell proliferation results. ns—no significance, * *p* < 0.05. (**D**) HT29-PTK7 ^V354M^ transfected cells showed upregulated CCND1 and CCNE protein expression, involved in cell cycle regulation. *PTK7^V354M^–PTK7 plasmid with the mutation V354M, PTK7^WT^–wild type PTK7 plasmid, pcDNA3–control vector*.

To investigate the impact of the *PTK7*^V354M^ variant on downstream targets influencing increased cell proliferation, we measured the gene expression profile of important components of cancer-related signaling pathways using qPCR. Our results showed down-regulation of *p53*, *p21*, and *CREB* mRNA levels in HT29-*PTK7*^V354M^ compared to HT29-*PTK7^WT^* cells, suggesting the involvement of mutated *PTK7* in suppressed transcription of those genes (Figure 4A).

### 2.7. PTK7^V354M^ Variant Enhances AKT and Suppresses CREB, p53 and p21 Protein Expression

Further analysis of the impact of the introduced *PTK7^V354M^* variant on protein expression levels of downstream targets involved in cancer-associated pathways revealed decreased protein expression of CREB, p53, and p21 in HT29-*PTK7*^V354M^ compared to HT29-*PTK7*^WT^ cells. Furthermore, our experiments resulted in increased expression of phosphorylated AKT protein (pAKT, S473) in HT29-*PTK7*^V354M^ compared to HT29-*PTK7*^WT^ cells. The described findings suggest that the investigated *PTK7*^V354M^ variant and the resulting overexpression of PTK7 protein may modulate signaling pathways involving AKT, CREB, p53, and p21, potentially leading to enhanced proliferation of HT-29 cells (Figure 4B).

### 2.8. AKT Inhibition Failed to Rescue the CREB Expression in PTK7V^354M^ Variant

As we found that CREB expression was reduced in HT29-*PTK7*^V354M^ cells while pAKT was increased, we inhibited the expression of AKT by using Perifosine to test whether CREB expression could be rescued or not. Rescue experiments were performed in two cell lines, HT29 and LS174T. As shown in Supplementary Figure S2, AKT was successfully inhibited in both cell lines, and we observed a trend towards restoration of CREB in LS174T cells.

### 2.9. PTK7^V354M^ Variant Increases Cell Cycle Progression and Upregulates Wnt Downstream Targets

To explore the underlying reason for increased cell proliferation, invasion, and migration, we examined the effects of HT29-PTK7^V354M^ on cell cycle progression. As illustrated in Figure 4C, HT-29 cells transfected with HT29-PTK7^V354M^ were observed less in the G0/G1 phase of the cell cycle but more in the G2/M phase, when compared to cells transfected with HT29-PTK7^WT^. These findings are consistent with down-regulation of *CREB*, *p53*, and *p21* and up-regulation of AKT, suggesting that the *PTK7^V354M^* variant is involved in cell-cycle regulation. We further quantified protein levels of CCND1 and CCNE involved in cell cycle regulation and found that they were up-regulated (Figure 4D).

Similar trends were observed for proliferation, cell cycle progression and migration in LS174T cells transiently transfected with *PTK7*^WT^ and *PTK7*^V354M^ (Supplementary Figure S3).

## 3. Materials and Methods

### 3.1. Population Recruitment

In several regions of Poland, population screening was performed mainly in years 2000–2014, in which questionnaires about cancer family history were collected. Persons with positive CRC family history were invited to regional genetic outpatient clinics, and their more detailed family histories were collected through detailed face-to-face interviews. Similarly, persons with negative cancer family history were interviewed. An average review took 20–30 min. Eligible individuals were asked to participate in the study. Altogether, 1705 unrelated familial CRC cases and 1674 healthy elderly individuals without family history of cancer provided a blood sample for genetic analyses and signed informed consent.

All families were screened for alterations in *APC*, the mismatch repair genes *MLH1*, *MSH2*, *MSH3*, large deletions in *EPCAM* and *POLE* p.Leu424Val, *POLD1* p.Ser478Asn and *NTHL1* p.Gln90* mutations and were found to be negative.

### 3.2. Colorectal Cancer Family

**Figure 5:**
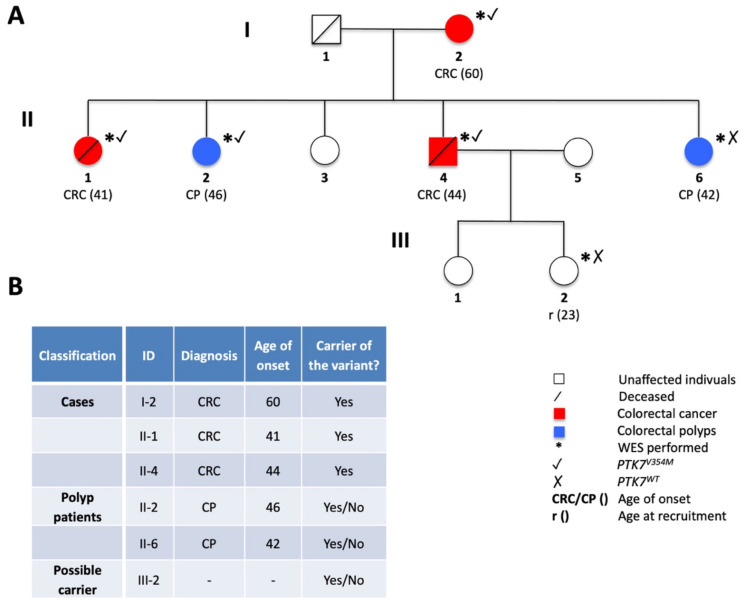
(**A**) The pedigree of the CRC-affected family carrying the *PTK7^V354M^* variant. (**B**) Tabular summary of family members including personal data and the carrier status of the cancer-causing mutation. *CRC—colorectal cancer, CP—colorectal polyps, WES—whole-exome sequencing, r—recruitment*.

For the present study, one of the Polish families with CRC aggregation was recruited, presenting 3 family members with CRC (I-2, II-1, II-4) and 2 with colorectal polyps (II-2, II-6), who agreed to participate in the study and provided a blood sample. Additionally, 1 unaffected family member (III-2) was sequenced, as represented in Figure 5A.

### 3.3. Validation Cohort

The 1704 familial CRC cases, not related to the whole-exome-sequenced family, and 1674 healthy elderly individuals were included in the validation cohort.

### 3.4. Ethical Permissions

The study was approved by the Bioethics Committee of the Pomeranian Medical Academy in Szczecin No: BN-001/174/05. All participating individuals signed informed consent.

### 3.5. Whole-Exome Sequencing

Peripheral blood samples were collected from affected and unaffected family members who agreed to participate in the study as well as from the validation cohort. Genomic DNA was isolated using a modified Lahiri and Schnabel method [29]. WES was performed using Agilent SureSelect V5 with UTR target capture kit on the Illumina HiSeq 2000 platform (Illumina, San Diego, CA, USA).

### 3.6. WES Alignment and Variant Calling

Raw sequencing reads were mapped to the human reference genome (GRCh37, assembly version Hs37d5) with BWA (version 0.6.2,Genome Research Limited, London UK) [30], and duplicates were marked using Picard (http://broadinstitute.github.io/picard/, accessed on 22 January 2020). Single nucleotide variants (SNVs) and small insertions/deletions (indels) from all family samples were called together using SAM tools [31] Platypus (version 0.8.1, Wellcome Centre for Human Genetics, Oxford UK) [32]. Variants were annotated with Gencode (version 19, Wellcome Sanger Institute, Hinxton, UK) gene definition using ANNOVAR (Center for Applied Genomics, Philadelphia, PA, USA) [33]. Furthermore, minor allele frequencies (MAFs) were accessed from 1000 Genomes, dbSNP and nonTCGA Exome Aggregation Consortium (ExAC) (version 0.3, Cambridge, MA USA) [34,35,36]. Variants with a quality score of greater than 20 and a coverage of greater than 5× and indels that passed all the Platypus internal filters were further evaluated for MAF. Variants with a MAF ≤ 0.1% with respect to the 1000 Genomes Project Phase 3 and non-TCGA ExAC data and variants with a frequency of <5% in the local dataset were deduced for further evaluation.

A pairwise comparison of shared rare variants among the cohort was performed to check for sample swaps and family relatedness.

### 3.7. Familial Segregation of the CRC Predisposing Variant

All family members were assessed for the probability of being a Mendelian case and thus of carrying the variant of interest. Cancer patients were defined as *cases* and were therefore expected to carry the mutation: the two affected siblings II-1 and II-4 who developed CRC at the relatively young age of 41 and 44 years, respectively, and their affected mother (I-2) with CRC at the age of 60 years. The other two siblings (II-2, II-6) were diagnosed with colorectal polyps (CP) in their 40s. Therefore, they also bear the risk of expressing a preliminary stage of CRC and were thus considered as *polyp patients*, possibly carrying the mutation. The descendant III-2 of one of the CRC-affected siblings (II-4) had not reached the earliest age of onset in this family at the time of family recruitment (23 years) and was hence defined as a *possible carrier* of the variant. All identified variants were filtered according to the described pedigree criteria, respectively summarized in Figure 5B.

### 3.8. Evaluation of the Deleterious Nature of Identified Variants Using FCVPPv2

All the rare coding variants identified by WES were evaluated using our in-house developed Familial Cancer Variant Prioritization Pipeline version 2 (FCVPPv2, DKFZ, Heidelberg, Germany), comprising non-synonymous SNVs and small indels [14]. 

Variants were ranked using the combined annotation-dependent depletion tool (CADD) v1.3, considering only the top 10 % of probable deleterious variants (PHRED-like CADD score ≥ 10) for further analysis [37]. In order to evaluate the evolutionary conservation correlating with the functional importance of a genomic position, the Genomic Evolutionary Rate Profiling (GERP ≥ 2.0), PhastCons (>0.3), and PhyloP score (≥3.0) were assessed [38,39]. Next, the intolerance of genes against genetic variation was considered using three intolerance scores (<0) based on allele frequency data from our in-house datasets, from ESP and ExAC [40,41]. Additionally, the Z-Score (>0) and pLI score (≥0.9) developed by ExAC consortium were included in our intolerance screening for missense and loss-of-function variants, respectively. Regarding non-synonymous and splice site SNVs, the deleteriousness of variants was evaluated by applying 10 different scoring systems and 2 meta-prediction tools derived from dbNSFP v3.0 (database for nonsynonymous SNPs’ functional predictions) [42]. The variants should reach a PHRED-like CADD-score of ≥ 10 and fulfill at least 2 out of 3 conservational scores, 60% of all intolerance scores, and 60% of all deleteriousness scores to be further considered in the analysis. Lastly, the top exonic candidates were further screened by considering the allele frequency in the non-Finnish European population in the latest version of the gnomAD Browser (https://gnomad.broadinstitute.org/, accessed on 25 January 2020) [20]. The potential of the variants as cancer drivers were predicted by Cancer Genome Interpreter (CGI, https://www.cancergenomeinterpreter.org/, BBGLab, Barcelona, Spain, accessed on 29 January 2020) [21], alteration frequencies according to cBioPortal (MSKCC, New York, NY, USA, accessed on 19 March 2020) [22] and by recent literature for reported gene–cancer relations and potentially cancer-related protein functions.

### 3.9. Confirmation of Familial Segregation by Sanger Sequencing

Exon 7 of the *PTK7* gene (ENST00000230419.4) was amplified from DNA of each family member (I-2, II-1, II-2, II-4, II-6 and III-2) by performing Polymerase Chain Reaction (PCR) with HotStarTaq DNA Polymerase (Qiagen, Venlo, Netherlands, #203205) at an annealing temperature of 56 °C. The primers were designed with Primer3 v.0.4.0 (http://bioinfo.ut.ee/primer3-0.4.0/, DKFZ-ZMBH Alliance, Heidelberg, Germany, accessed on 12 February 2020): PTK7 forward 5′-GTTTTTACTCAGCCGCTTGG-3′, reverse 5′-AAGCCTCTGTCCATCACACC-3′. PCR amplicons were checked by gel electrophoresis and purified with ExoSAP purification kit according to the manufacturer’s instructions. Sequencing reaction was performed with the BigDye Terminator v3.1 Ready Reaction Cycle Sequencing kit (Thermo Fisher Scientific, Waltham, MA, USA, #4337455) and the electrophoretic profiles of *PTK7* sequences were analyzed manually.

### 3.10. Screening of Familial CRC Index Cases and Healthy Individuals by Taqman Assay

For screening a large cohort of familial CRC cases not related to the studied family, the *PTK7* variant was screened in 1704 familial CRC cases and 1674 healthy elderly individuals, both from Poland, using a custom-made Taqman assay.

### 3.11. Plasmid Preparation and Cell Culture 

pcDNA3-PTK7-VSV (#65250) was purchased from Addgene (Watertown, MA, USA) and used in functional experiments as the wild type *PTK7* plasmid (*PTK7^WT^*). Mutant *PTK7* plasmid (*PTK7^V354M^*) was created using QuikChange II XL Site-Directed Mutagenesis Kit (Agilent Technologies, Santa Clara, CA, USA, #200521) and primers (forward: 5′-GCTCCCACCACATGCTGGGCTCTGG-3′ and reverse: 5′-CCAGAGCCCAGCATGTGGTGGGAGC-3′) designed based on Agilent QuikChange Primer Design (https://www.agilent.com/store/primerDesignProgram.jsp, accessed on 12 February 2020). After confirmation of both plasmids by Sanger sequencing, XL10-Gold Ultracompetent Cells (Agilent Technologies, Santa Clara, CA, USA, #200314) were used for transformation and PureLink™ HiPure Plasmid Midiprep Kit (Thermo Fisher Scientific, Waltham, MA, USA, #K210004) was utilized for plasmid extraction according to the manufacturer’s instructions. Human colon cancer cell line HT-29 (kind gift from Peter Krammer’s lab, DKFZ, Heidelberg, Germany) and colorectal adenocarcinoma cell line LS174T (AddexBio, San Diego, CA, USA, #C0009013) were cultured in RPMI 1640 media supplemented with 10% FBS, 2 mM L-glutamine (Sigma-Aldrich, St. Louis, MO, USA, #51411C) and used in the experiments. After 48 h of transfection, LS174T cells were analyzed for functional output and stably transfected cells were selected for HT-29 cells by using G418 (final concentration 400 ug/mL: pcDNA3 (HT29-pcDNA3), *PTK7^WT^* (HT29-*PTK7^WT^*) and *PTK7^V354M^* (HT29-*PTK7^V354M^*)). Medium containing G418 was changed every 2–3 days until the surviving colonies were pooled (polyclonal line) and used for single-cell sub cloning. Monoclones were achieved by serial dilution and used for further experiments.

### 3.12. Cell Proliferation Assays

HT-29 cells were seeded in 24-well plates and 24 h later transfected with either 150 ng of *PTK7^WT^*, *PTK7^V354M^*, or pcDNA3 vector as a negative control. Cells were washed with PBS and trypsinized. After excluding the dead cells with trypan blue, viable cell numbers were determined by cell counting with the hemocytometer under a 10× objective at six different time points: day 0, 1, 2, 3, 4, and 5. Number of viable cells and respective proliferation curves were compared between HT29-*PTK7^WT^* and HT29-*PTK7^V354M^* cells.

### 3.13. Quantitative Polymerase Chain Reaction

RNA was isolated from cells (HT29-pcDNA3, HT29-*PTK7^WT^*, HT29-*PTK7^V354M^*) using Trizol reagent and purified with sodium acetate. For cDNA synthesis, ProtoScript First Strand cDNA Synthesis kit (New England Biolabs, Ipswich, MA, USA, #E6300S) was used according to the manufacturer’s instructions. Quantitative real time Polymerase Chain Reaction (qPCR) was performed using QuantiFast^®^ SYBR^®^ Green PCR (Qiagen, Venlo, The Netherlands, #204054). Primers for downstream targets (*ERK*, *CREB*, *p53*) and the housekeeping gene *HPRT* (Hypoxanthin-Guanin-Phosphoribosyltransferase) as an internal control are provided with respective primer sequences in Supplementary Table S1. Relative gene expression was calculated with the 2^ΔCT^ method and compared between HT29-*PTK7^WT^* and HT29-*PTK7^V354M^* cells.

### 3.14. Western Blot

Protein lysates were prepared and quantified by means of Pierce™ BCA Protein Assay Kit (Thermo Fisher Scientific, Waltham, MA, USA, #23225). NuPAGE™ 4–12% Bis-Tris Protein Gels and the respective running buffer (Thermo Fisher Scientific, Waltham, MA, USA; #NP0321PK2, #NP0001) were used for separation of 20 μg of each protein sample. Blotted membranes were blocked with 2% milk for 1 h, incubated overnight at 4 °C with primary antibody and subsequently for 1 h at room temperature with the respective HRP-conjugated secondary antibody, and diluted in 5% milk. Blots were developed using Amersham ECL Western blotting detection kit (GE Healthcare Life Sciences, Chicago, IL, USA, #RPN2108). Glyceraldehyde 3-phosphate dehydrogenase (GAPDH) and β-actin antibodies were used for loading quantity control. All probed antibodies are summarized in Supplementary Table S2 with respective product details, dilution buffers and dilution factors.

### 3.15. Wound Healing Assay

Wound-healing assays were used to measure cell migration. The single-cell clones of HT-29 and transiently transfected LS174T cells were seeded in the Culture-Insert 2 Well in *µ*-Dish (ibidi GmbH, Martinsried, Germany, #81176) at a density of 3 × 10^4^ cells per well. Then, the culture-insert was removed, and the media were exchanged with fresh serum-reduced media (2% FBS). The plate was photographed at different time points, and the insert space was quantified by imageJ, v1.53.

### 3.16. Invasion Assay

One hundred microliters of the diluted Matrigel matrix (Corning, Wiesbaden, Germany, #356234) was carefully added to the center of each Transwell^®^ insert (Corning, Wiesbaden, Germany, #3464) for invasion assays and incubated at 37 °C for 1 h to allow the Matrigel matrix to form a gel. Then, 200 μL of serum-free medium per single cell clone of HT-29 (2.5 × 10^5^/mL) was seeded into inserts and put in cell culture wells. The media were removed after 24 h and washed with PBS. Cells were fixed with 4% PFA, and the remaining buffer in upper chamber was removed, washed twice with PBS and the membrane was stained in 0.1% crystal violet for 30 min at room temperature. Cells were viewed underneath an inverted microscope and six visual fields were randomly chosen to calculate the number of migrated cells.

### 3.17. Cell Cycle Analysis

HT-29 and LS174T cells were collected and fixed by adding freshly prepared 70% ice-cold ethanol dropwise to the cell pellet and vortexed gently. After the pellet was washed, propidium iodide (50 μg/mL) and RNase A (1 μg/μL) were added and cell cycle analysis was performed in Canto two-laser FACS analyzer (BD, Franklin Lakes, NJ, USA). Doublets were excluded by applying the following gates: SSC-W vs. SSC-A and PI-W vs. PI-A. FACS data were analyzed using Flowjo X 10.0.7 (Tree Star, Ashland, KY, USA).

### 3.18. AKT Inhibition

HT-29 and LS174T cells were seeded in 6-well plates 24 h before plasmid transfection with serum-free media. Media was removed after 7 h of transfection and exchanged with fresh media containing the AKT inhibitor-Perifosine (Cell Signaling Technology, Denver, MA, USA, #14240S). 24h later the protein was collected and the targets of interest were detected by Western blot.

All the experiments were performed in triplicates and repeated twice unless otherwise stated.

## 4. Discussion

The current state of research indicates very few germline variants for the *PTK7* gene that have been associated with the development of any disease. A recent genome-wide linkage analysis of Swedish CRC families identified a candidate risk variant in the *PTK7* gene (A785V) [43]. Additionally, germline variants in *PTK7* have been associated with neural tube defects [44]. Applying our in-house developed FCVPPv2 on a family with CRC aggregation, we identified a novel germline variant (V354M) in the *PTK7* gene, encoding a catalytically inactive tyrosine kinase receptor. This finding led us to explore a potential association between PTK7 and CRC predisposition and to screen a large cohort of 1704 familial CRC index patients and 1674 healthy elderly individuals, both from Poland, using custom-made Taqman assay for the V354M variant. In the familial CRC index cases, we identified an additional case with the same variant but did not find any in the controls. Our finding is the second report of a germline variant in *PTK7* in two families with a history of CRC.

Several studies have reported up-regulation of PTK7 protein in a number of malignancies including CRC [17,45,46,47,48,49,50]. PTK7 has been shown to display phenotypes of both oncogene and tumor suppressor gene, depending on the cell type and its interaction partners, but to mostly act as an oncogene in CRC [17,48,51,52,53,54,55,56,57,58,59]. Thus, a better understanding of the molecular function of PTK7 in cancer development in general and cancer-related pathways in particular requires further experimental investigation. In our study, the high conservation of the affected region, the high alteration frequency of *PTK7* in several cancers, and its postulated oncogenic function based on recent literature prompted us to prioritize the *PTK7*^V354M^ variant for further functional validation.

By analyzing the functional impact of the identified *PTK7^V354M^* variant in human CRC cells, we were able to propose molecular mechanisms potentially influenced by the mutated PTK7 protein in CRC susceptibility. We observed increase of PTK7 protein expression induced by the variant, which may be explained by altered protein–protein interactions. Since binding of the canonical Wnt ligands (e.g., Wnt3a, Wnt8) at the extracellular region of the PTK7 protein mediated by Fz7 (frizzled class receptor 7) has been reported to initiate endocytosis and lysosomal degradation of PTK7, the induced amino acid substitution affecting one of the extracellular Ig loop domains may interfere with ligand binding and thus inhibit PTK7 degradation [60,61]. Of course, further potential mechanisms resulting in enhanced protein expression (e.g., via affecting the transcriptional machinery or mRNA stability) and protein stability (e.g., due to altered protein folding) have to be taken into account as a possible reason for PTK7 overexpression induced by the variant. By affecting one of the extracellular Ig loops, the variant may not only show an impact on PTK7 stability and degradation but may further influence important extracellular signaling interactions. Binding of Wnt3a and Wnt8 has been reported to lead to inhibition of canonical Wnt signaling in the Xenopus double axis [60,61]. Assuming an impaired binding of those ligands to the extracellular PTK7 region due to the studied variant, Wnt3a and Wnt8 may instead interact with Wnt receptor complexes and thus activate canonical Wnt signaling.

Further interactions of the extracellular region of PTK7 have been documented for non-canonical Wnt ligands. Using truncated forms of the PTK7 protein in co-immunoprecipitation assays in HEK293T cells, Martinez et al. have shown that extracellular loops 4–7 of PTK7 are partially required for binding to Wnt5a ligand promoting phosphorylation of JNK and cell movement [62]. In addition to the direct interaction with Wnt5a, the whole extracellular domain of the PTK7 protein covering Ig loops 1–7 has been reported to form a receptor complex with ROR2 (receptor tyrosine kinase-like orphan receptor 2), an active tyrosine kinase receptor, and Wnt5a-dependent trigger of non-canonical Wnt signaling [62,63]. However, the functional role of Wnt5a in CRC development has been described in contradictory ways over the past years [64], and little is known about specific interaction sites located in particular Ig loops. Thus, further investigation is needed for better understanding of how the studied variant may affect the potential ligand binding in the course of canonical as well as non-canonical Wnt signaling.

In addition to the described effects on PTK7 protein levels, we showed that introduction of the *PTK7^V354M^* variant into cells increased their migration, invasion, and cell-cycle progression, and it enhanced phosphorylation of the AKT protein, an oncoprotein involved in multiple cellular processes such as cell proliferation, survival, growth, and angiogenesis. Similar results were reported in in vitro studies using esophageal squamous cell carcinoma cells. Knockdown of PTK7 has been reported to lead to decreased AKT phosphorylation and activation [54], whereas another study observed initially enhanced and subsequently suppressed AKT phosphorylation due to PTK7 overexpression, indicating a biphasic regulation [65]. Nevertheless, both studies showed an association of PTK7-induced AKT phosphorylation with increased cell proliferation, migration, and invasion, similar to the findings of our in vitro experiments [54,65]. In vascular endothelial cells, AKT phosphorylation was further reported to be a downstream signal of the VEGF-A-stimulated receptor complex VEGF-R1 and PTK7, resulting in increased angiogenesis [66]. Besides potentially affecting the VEGF signaling pathway, *PTK7^V354M^*-induced AKT phosphorylation may be explained by additional molecular mechanisms that still have to be explored.

Activated AKT in turn might constitute an important effector protein of PTK7 responsible for the proposed carcinogenic impact of the variant: by signaling to various downstream targets including mTOR, forkhead transcription factors, or GSK-3, it can affect several molecular pathways including the Wnt signaling pathway. AKT inactivates GSK-3 by phosphorylation, resulting in decreased *β*-catenin degradation. Thus, *β*-catenin can translocate to the nucleus and activate gene expression of several Wnt targets, contributing to colorectal carcinogenesis [67].

Furthermore, we observed *PTK7^V354M^* induced down-regulation of mRNA and protein levels of cAMP response element binding protein (CREB), in accordance with the downstream signaling effects of full-length PTK7 protein in cancer cells described by Golubkov et al. The authors proposed inhibition of ERK which is known to regulate CREB protein activity by phosphorylating it at serine residue 133 [68]. Since this phosphorylation has been reported to prevent CREB from heterodimerization and thus protein degradation, ERK could additionally be involved in the regulation of CREB protein levels [69]. However, our results did not show that *PTK7^V354M^* induced deregulation of ERK transcription or phosphorylation (data not shown) but demonstrated that CREB suppression already occurred at mRNA level. We also tested the dependency of *PTK7^V354M^*-induced down-regulation of CREB on AKT, a known regulator of CREB protein. Since we observed a trend towards restoration of CREB upon inhibition of AKT in LS174T cells, AKT may play a mediating role in CREB suppression, induced by the variant. As we were not able to reproduce these results in HT-29 cells, the involvement of additional repressing mediators and mechanisms affecting *CREB* expression may be assumed and remains to be elucidated. Since CREB transcription factor in turn has been reported to transactivate *p53* gene expression and thus to induce apoptosis programs, the down-regulation of *p53* gene, and its downstream target p21 detected in our experiments may be explained as a downstream effect of CREB suppression [70].

P53 and p21 are known cell-cycle regulators inhibiting G1/S and G2/M transition upon DNA damage. Thus, the observed down-regulation of p53 and p21 proteins may facilitate G1/S transition and may explain the decrease of mutated HT-29 cells in G0/1 phase. This is further supported by our findings of increased cyclin D1 (CCND1) and cyclin E (CCNE) expression, both downstream targets of p21 and inductors of G1/S cell cycle transition. However, we did not observe a significant difference in cells in S phase but a shift of mutated cells towards G2/M phase compared to cells transfected with wild-type *PTK7*. Summarizing our results, *PTK7^V354M^* showed a significant impact on cell-cycle progression, indicating again its carcinogenic potential.

Since our study is the second report of a *PTK7* germline variant in familial CRC, to our knowledge, the present results not only support the establishment of the oncogenic function of PTK7 in CRC but also suggest the implementation of *PTK7* as an oncogene in genetic inheritance of familial colorectal malignancies. Despite the overall observed low frequency of the *PTK7^V354M^* variant, our findings may be of importance for the studied family, for genetic screening of unaffected family members, and for the development of personalized therapeutic regimens for affected members. The relevance of the *PTK7^V354M^* variant for other individuals at risk of developing familial CRC is further supported by the finding of an additional carrier of exactly the same variant in the *PTK7* gene, identified in an unrelated family of three CRC patients from Poland. Although the *PTK7^V354M^* variant was identified in two CRC families, we could only confirm the segregation of the variant with the disease in the whole-exome-sequenced family, since a DNA sample was only available for the index case of the second family, which is a limitation of our study. Other limitations include the absence of clinical or pathological features of the CRC patients, their tumors, and their polyps. We also did not have access to the tumor samples to evaluate loss-of-heterozygosity, although in the case of an activating variant, a change in one allele may be enough to have an impact on the activity of the encoded protein. However, more replication studies in independent cohorts and in vivo experiments are warranted to take these results to the next level.

In conclusion, we propose *PTK7* as a potential susceptibility gene in familial CRC, thus contributing to the exploration of the remaining genetic burden of familial colorectal malignancies. The result tends to reinforce the view that the germline architecture of CRC is dominated by the “first-wave” mismatch repair genes of high clinical importance, supplemented by the much rarer “second- and next-wave” genes (such as *PTK7*) and the many low-penetrant genes.

## Figures and Tables

**Table 1:** Exonic variants prioritized in the analyzed CRC family. Chromosomal positions, classifications, pedigree segregation, allele frequencies, PHRED-like CADD scores, conservational scores, and the percentage of positive intolerance and deleteriousness scores are summarized for each variant. Respective protein functions derived from Genecards are included [19].

Gene Name	Variant	Exonic Classification	Pedigree Segregation	Allele Frequency		CADD SCORE	Conservational Scores			Deleteriousness Scores * (%)	Intolerance Scores (%)	Amino Acid Change	Protein Function
				ExAC	gnomAD NFE		GERP++	PhyloP	PhastCons				
**ADAMTS10**	19_8670022_C_T	nonsyn SNV	I-2, II-1, II-2, II-4, II-6	1.92E-05	8.95E-06	32	5.33	7.263	1	66.67	80	V104M	Connective tissue organization, coagulation, inflammation, arthritis, angiogenesis, cell migration
**C2orf42**	2_70387896_G_C	nonsyn SNV	I-2, II-1, II-4, II-6	.	.	23.5	2.33	1.849	1	83.33	60	S459R	No data available
**GNA13**	17_63049685_T_C	nonsyn SNV	I-2, II-1, II-2, II-4	2.21E-04	3.33E-04	22.3	5.42	3.986	1	75	60	I149V	GPCR signaling, RhoA regulation pathway
**PTK7**	6_43100257_G_A	nonsyn SNV	I-2, II-1, II-2, II-4	0	0.000	25.3	4.14	4.217	1	66.67	100	V354M	Wnt signaling pathway, cell adhesion, migration, polarity, proliferation, actin cytoskeleton reorganization, apoptosis
**RSBN1L**	7_77407669_G_A	nonsyn SNV	I-2, II-1, II-4	0	0.000	35	5.94	7.575	1	100	80	R603H	Modulation of chromosome architecture by histone demethylation.
**TNIP1**	5_150431736_C_T	nonsyn SNV	I-2, II-1, II-2, II-4	1.86E-05	0.000	19.16	5.19	4.539	0.999	66.67	60	E238K	Autoimmunity, tissue homeostasis

non-syn—non-synonymous; NFE—Non-Finnish European population; PP—predicted passenger. * Deleteriousness scores: Following predictions given by deleteriousness scores were considered as favorable in our analysis: SIFT—Damaging (D); Polyphen2_HumDiv, Polyphen2_HumVar—Probably damaging (D) & Possibly damaging (P); LRT—Deleterious (D); MutationTaster—Disease causing (D) & disease causing automatic (A); MutationAssesor—High (H) & medium (M); FATHMM—Damaging (D); MetaSVM—Damaging (D); MetaLR—Damaging (D); VEST3 ≥ 0.5; PROVEAN—Damaging (D); Reliability Index ≥5.

## Data Availability

Unfortunately, for reasons of ethics and patient confidentiality, we are not able to provide the sequencing data into a public database. The WES data generated in this study are available from the corresponding author upon request.

## Supplementary Materials

Supporting information can be downloaded at: https://www.mdpi.com/article/10.3390/ijms23031295/s1.
