# Supplementary material for: Whole-Exome Sequencing Identifies a Novel Germline Variant in PTK7 Gene in Familial Colorectal Cancer
Source: Int J Mol Sci. 2022 Jan 24;23(3):1295. doi: 10.3390/ijms23031295 (PMC8836109; doi:10.3390/ijms23031295)
Supplement: Supplementary file 1 [file ijms-23-01295-s001.zip › ijms-1545025-supplementary.pdf]

Supplementary Materials

Supplementary Table S1. List of qPCR primers

Gene	Forward primer sequence	Reverse primer sequence
**CREB**	Quantitect primer assay purchased from Qiagen	
**ERK**	ACACCAACCTCTCGTACATCGG	TGGCAGTAGGTCTGGTGCTCAA
**HPRT**	Quantitect primer assay purchased from Qiagen	
**p53**	Quantitect primer assay purchased from Qiagen	
**PTK7**	GAGATGACGCTGGCAACTACAC	CGCTCTGGTTCCACTTTGAAGG
**P21**	Quantitect primer assay purchased from Qiagen	

Supplementary Table S2. List of primary and secondary antibodies.

PRIMARY ANTIBODIES

Protein target	Host	Cat Number	Company	Dilution buffer	Dilution factor
**pAKT (Ser473)**	Rabbit	4060	Cell Signaling Technology	5% BSA in TBST	1:2000
**PanAKT**	Rabbit	4691s	Cell Signaling Technology	5% milk in TBST	1:1000
**P21**	Rabbit	Ab109199	Abcam	5% milk BSA in TBST	1:1000
**CREB**	Mouse	86B10	Cell Signaling Technology	5% milk in TBST	1:1000
**ERK**	Rabbit	9102	Cell Signaling Technology	5% milk in TBST	1:1000
**GAPDH**	Mouse	CB1001	Millipore	5% milk in TBST	1:3000
**Anti-alpha Tubulin**	Rabbit	ab4074	Abcam	5% milk in TBST	1:1000

**p53**	Mouse	1C12	Cell Signaling Technology	5% milk in TBST	1:1000
**PTK7**	Goat	AF4499	Novus biologicals	5% BSA in TBST	1:200
**SECONDARY ANTIBODIES**					
**Species reactivity**	**Host**	**Catalog number**	**Company**	**Dilution buffer**	**Dilution factor**
**Goat**	Donkey	Sc-2020	Santa Cruz Biotechnology	5% milk in TBST	1:5000
**Mouse**	Horse	7076s	Cell Signaling Technology	5% milk in TBST	1:5000
**Rabbit**	Goat	7074s	Cell Signaling Technology	5% milk in TBST	1:5000

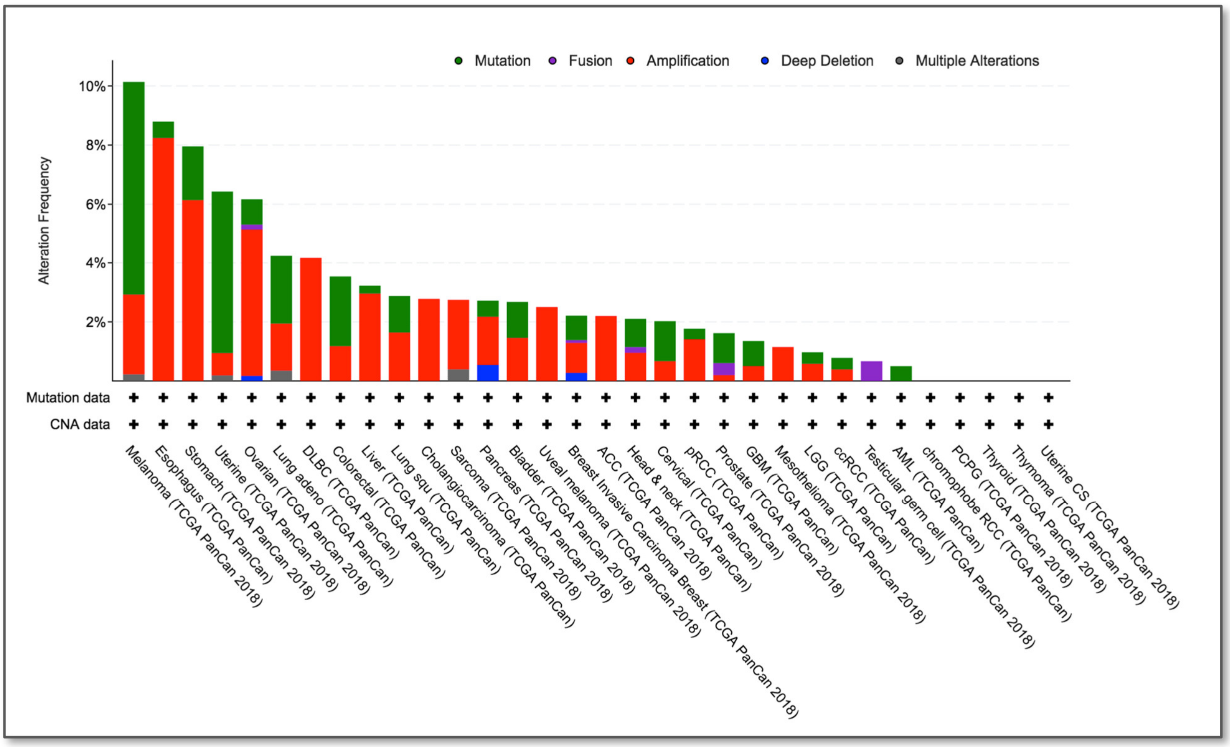

Supplementary Figure S1. PTK7 alterations in cancers. Histogram of alteration frequencies according to TCGA PanCancer data was extracted from cBioPortal (www.cbioportal.org) on 15th of March 2020.

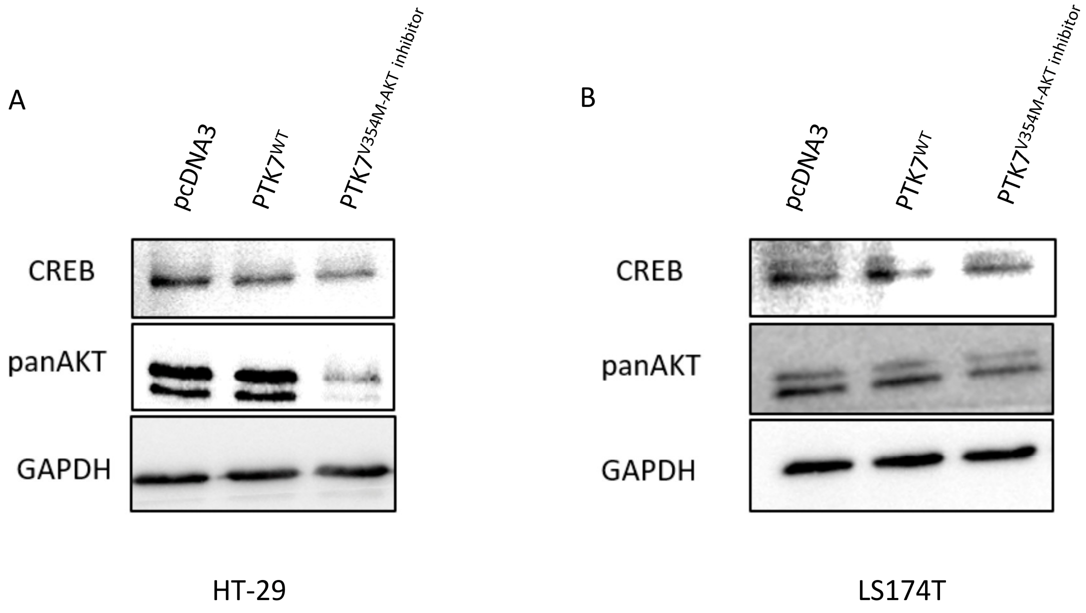

Supplementary Figure S2. **Effect of AKT inhibition on CREB expression in *PTK7V*^{354M} variant**

Inhibition of AKT expression by using Perifosine did not rescue the CREB expression in (A) HT-29 and (B) LS174T cells transfected with *PTK7*^{V354M}. AKT was inhibited successfully in both cell lines, but there was only a trend towards restoration of CREB in LS174T cells.

A

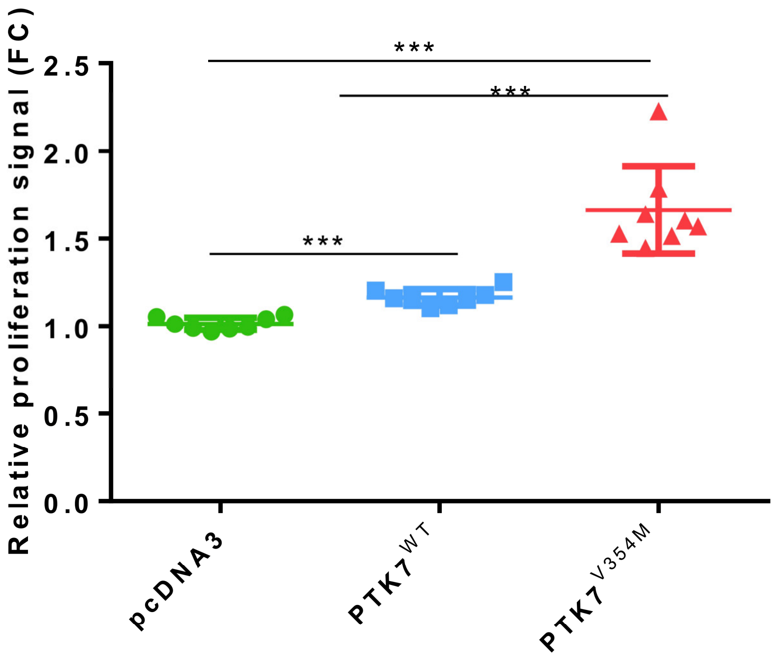

Relative proliferation signal (FC)
2.5
2.0
1.5
1.0
0.5
0.0
***
***
***
pcDNA3
PTK7WT
PTK7V354M

B

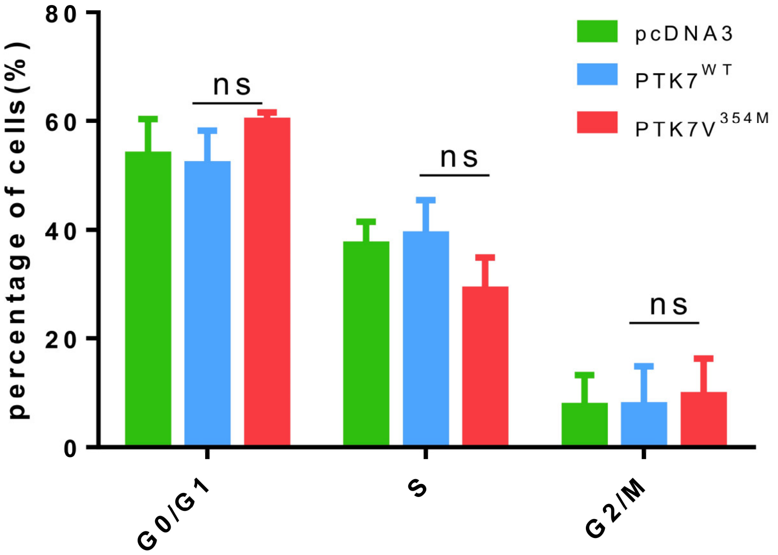

percentage of cells(%)
80
60
40
20
0
ns
ns
ns
pcDNA3
PTK7WT
PTK7V354M
G0/G1
S
G2/M

C

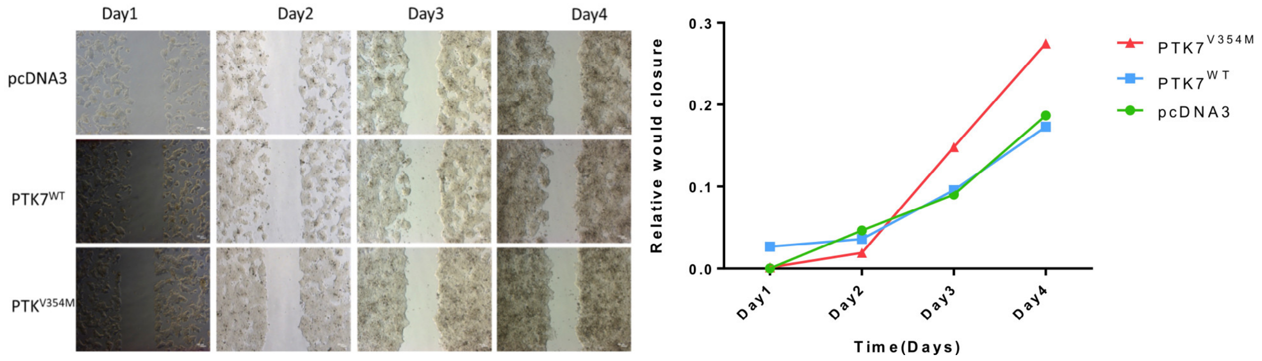

Supplementary Figure S3. ***PTK7*^{V354M} variant increases cell proliferation, cell cycle progression and migration.** (**A**) Proliferation assay showed significantly increased viable cell numbers of LS174T-*PTK7*^{V354M} compared to LS174T-*PTK7*^{WT} cells. Significance levels are included for each time point. *** - $p \leq 0.001$. (**B**) LS174T ^{V354M} transfected cells resulted in significant cell cycle progression. (**C**) Representative image of migrated LS174T cells after overexpression of *PTK7*^{WT} *and* LS174T-*PTK7*^{V354M}.
